# Parental major life events before or during pregnancy and autistic behaviors among preschool children

**DOI:** 10.1038/s41598-025-22094-z

**Published:** 2025-10-31

**Authors:** Weiying Liu, Yulan Wu, Dongyan Wen, Huiting Chen, Jinming Liu, Shuang Wu, Jiarong Lin, Zizi Liu, Xuanshu Wang, Lvping Li, Feixiang Zeng, Ruoqing Chen, Yu Jin

**Affiliations:** 1 Department of Epidemiology, School of Public Health (Shenzhen), Shenzhen Campus of Sun Yat-sen University, Shenzhen, 518107 China; 2 Department of Maternal and Child Health, School of Public Health, Sun Yat-Sen University, Guangzhou, 510000 China; 3 Child Developmental and Behavioral Center, The Third Affiliated Hospital of Sun Yat-sen University, Guangzhou, 510630 China; 4 Department of Rehabilitation, The Third People’s Hospital of Huizhou City, Huizhou, 516000 China; 5 Institute of Environmental Medicine, Karolinska Institutet, SE-171 76 Stockholm, Sweden

**Keywords:** Autistic behaviors, Preschool children, Major life events, Alcohol consumption, Smoking, Developmental neurogenesis, Risk factors, Autism spectrum disorders

## Abstract

In this study, we aimed to examine whether parental major life events before or during pregnancy were associated with autistic behaviors in preschoolers, and whether alcohol use or smoking modified these associations. This study included 18,664 children aged 3–6 years in southern China. Parents reported seven types of major life events before or during pregnancy. Autistic behaviors were assessed by the Clancy Autism Behavior Scale. Logistic regression was applied to evaluate the associations of parental major life events with the risk of autistic behaviors, along with the modifying effects of alcohol consumption and smoking. This study found that paternal exposure to major life events before or during pregnancy was associated with an increased risk of autistic behaviors. Maternal exposure to major life events during pregnancy was associated with a higher risk of autistic behaviors. The associations between paternal pre-pregnancy major life events and autistic behaviors were stronger when either parent consumed alcohol before pregnancy. No modifying effect was shown for smoking. Findings from this study highlighted the importance of monitoring parental stress and alcohol use before and during pregnancy to improve children’s neurodevelopmental outcomes.

**Supplementary Information:**

The online version contains supplementary material available at 10.1038/s41598-025-22094-z.

## Introduction

Autism Spectrum Disorder (ASD), known as a neurodevelopmental disorder, is primarily defined by deficits in social communication, along with repetitive behaviors and restricted interests^1^. Currently, the global prevalence of ASD is approximately 1%^2^. A nationwide population-based study reported a 0.7% prevalence in 6 to 12 years old children in China^3^, highlighting ASD as a significant public health concern for children and families^4^.

Substantial evidence has highlighted the critical role of maternal major life events occurring before or during pregnancy in the development of ASD or autistic behaviors in children. A case–control study comparing 115 autistic children and 112 typically developing children found that mothers of autistic children experienced significantly higher frequencies of both negative life events, such as high debt, frequent marital conflict, and positive life events, such as major job progress, starting or finishing education during pregnancy^5^. A retrospective study in 174 autistic children found that exposure to prenatal major life events, such as separation or divorce, involuntary job loss, residential move, death of a close friend or relative, or other stressful events, was associated with more severe autistic symptoms and suboptimal communication level^6^. A study including 65,931 preschool children found that prenatal maternal stressful events, particularly during the second trimester, were associated with an increased risk of autistic-like behaviors in preschoolers^7^. However, the impact of paternal major life events on autistic behaviors in children remains under-researched but is equally important to consider given that fathers contribute to offspring development through both biological (e.g., sperm epigenetics) and psychosocial (e.g., paternal caregiving and household stress) pathways^8^.

The mechanisms underlying the associations of parental preconception and prenatal major life events with children’s autistic behaviors are yet to be fully understood. Potential biological mechanisms may involve stress-induced alterations in hypothalamic–pituitary–adrenal (HPA) axis function, prenatal immune dysregulation, and epigenetic modifications, which collectively disrupt fetal brain programming and increase susceptibility to autistic behaviors^9–11^. It is also important to consider the lifestyle changes resulting from stress coping strategies, which may include increasing alcohol consumption and smoking. Alcohol consumption and smoking, both known to negatively affect fetal programming^12^, often coincide with the experience of major life events^13,14^. These lifestyle factors have been recognized as significant concerns for children’s neurodevelopment^15^. However, it remains unclear whether they modify the impact of major life events on children’s autistic behaviors.

This population-based study aimed to assess the associations between parental major life events before or during pregnancy and the risk of autistic behaviors in preschool children. Additionally, the study explored the potential role of parental alcohol consumption or smoking before or during pregnancy in modifying the studied associations.

## Methods

### Study design and participants

This study was embedded in a population-based autism screening program conducted in the Huicheng district, Huizhou City, Guangdong, China, from June 2022 to June 2023. The screening program covered 111 kindergartens in the Huicheng district, with invitations extended to 28,027 children. The screening procedure required parents of preschool-aged children to complete a comprehensive electronic questionnaire consisting of 116 items, which collected demographic information, autism screening scale data, and information about child growth and development. Eventually, the study included 18,664 singleton children aged 3–6 years whose parents consented to participate and completed the electronic questionnaires. Written informed consent was obtained from the parents of all participating children. All data were anonymized and de-identified before analysis.

### Exposure

The primary exposure was parental major life events before or during pregnancy. These events were assessed using four multiple-choice questions, evaluating whether either parent had experienced such circumstances (Supplementary Methods). Fathers reported major life events during the three months before the mother’s pregnancy, whereas mothers reported events over the entire year prior. Both parents also reported major life events during the mother’s pregnancy. Response options included marriage, divorce, relocation, job changes, severe illness or death of family members or close friends, others, or none. Based on these responses, we created dichotomous variables: “with/without major life events” separately for the pre-pregnancy and pregnancy periods for each parent. We further classified life events into positive, neutral, or negative categories based on their inherent nature. Marriage and promotion were considered positive life events, while divorce, demotion, and the experience of severe illness or the death of family members or close friends were considered negative life events. Residential relocation and other types of major life events were regarded as neutral, given their context-dependent nature.

### Outcome

Autistic behaviors were evaluated using the Chinese version of the Clancy Autism Behavior Scale (CABS), a tool intended to help parents assess and measure autistic behaviors in children^16^. The Chinese version of CABS consists of 14 items that cover different behavioral manifestations of ASD, assessing the frequency of these behaviors observed in the past month. Each item offers three options: “never” (0 score), “occasionally” (1 score), and “often” (2 scores). The total score results in a range from 0 to 28. Children with autistic behaviors are identified as those scoring 14 or above.

### Covariates

Variables known to be related to parental major life events, as well as autistic behaviors of children, were considered potential confounders (Supplementary Figures S1 and S2). These included: maternal factors (age at delivery, educational level, occupation, mode of conception, parity, diseases and medication use during the three months before pregnancy), paternal factors (age at child’s birth, educational level, occupation, diseases and medication use during the three months before the mother’s pregnancy), and children’s sex^17,18^. Mode of conception was categorized into “natural conception” and “assisted reproductive technology”. Parity was categorized into 1, 2, and 3 times or above. Parental age was categorized as < 25, 25 to 29, 30 to 34, 35 to 39, and ≥ 40^19^. Educational levels were classified as “senior high school or below” and “undergraduate or above”. Based on the commonalities and prevalence of occupations, mothers’ occupations were categorized into five groups: professionals, administrative and service workers, freelancers, stay-at-home mothers, and others; fathers’ occupations were categorized as professionals, administrative and service workers, freelancers, and others (Supplementary Table S1). Paternal and maternal diseases and medication use during the three months before the mother’s pregnancy were dichotomized as “yes/no”. Children’s sex was recorded as boys and girls.

To estimate the potential modifying effect of parental alcohol consumption and smoking on the studied associations, data on the alcohol consumption and smoking status of both parents before or during the mother’s pregnancy were utilized. Mothers reported whether they had consumed any alcohol in the three months before or during pregnancy, while fathers reported any alcohol consumption in the three months before pregnancy. Both parents reported whether they had smoked in the three months before or during pregnancy. All variables were categorized as “yes/no”.

### Statistical analyses

The rate of autistic behaviors in preschool children was calculated and presented as the number of children exhibiting autistic behaviors per thousand. Descriptive analyses were conducted to show the distribution of parental and child characteristics across parental exposure to major life events during the specified periods. We used logistic regression to assess the association between parental major life events before or during pregnancy and the risk of autistic behaviors in children. The results are presented as odds ratios (ORs) with 95% confidence intervals (CIs). Multivariable models were adjusted for maternal age at delivery, educational level, occupation, mode of conception and parity, paternal age at child’s birth, educational level, occupation, and children’s sex. In the analysis for parental major life events during pregnancy, we additionally adjusted for parental diseases and use of medication during the three months before mother’s pregnancy. To explore the potential joint effects of paternal and maternal exposures, we further created two joint exposure variables for the pre-pregnancy period and the pregnancy period. Each variable was classified into four categories: no, only father, only mother, and both parents exposed to major life events during the specified periods. To examine the cumulative burden of major life events, we also created variables indicating the numbers of major life events (0, 1, ≥ 2) for both parents during the specified periods.

Additionally, we performed analyses stratified by parental alcohol consumption and smoking status before or during pregnancy, and children’s sex. Formal tests of statistical interaction were used to evaluate whether these factors potentially modified the associations observed.

Given the maximum proportion of missing values in the variables studied was 12.5% (for parity), multiple imputation was conducted using the random forest method. Ten imputed datasets were generated, with 50 iterations performed for each dataset. A two-tailed P value less than 0.05 was regarded as statistically significant. The associations mentioned above were further evaluated in the imputed dataset. StataMP version 17.0 (Stata Corp LP, College Station, TX, USA) and R version 4.4.1 (R Foundation for Statistical Computing, Vienna, Austria) were used to conduct data preparation and statistical analyses mentioned above.

## Results

Among the 18,664 children studied, 1422 (76.2 per 1000 children) had autistic behaviors. A total of 2248 children (12.04%) were born to fathers who experienced major life events before the mother’s pregnancy, and 2160 children (11.57%) were born to fathers with such exposure during pregnancy. Similarly, 3011 children (16.13%) and 2121 children (11.36%) were born to mothers who experienced major life events before or during pregnancy, respectively. Parents who experienced major life events before or during pregnancy were more likely to be younger at the child’s birth, with higher educational levels, working in administrative and services industries, having diseases and medications during the three months before pregnancy (Tables 1 and 2).

**Table 1:** Characteristics of the study population according to paternal exposure to major life events.

Characteristics	Paternal exposure to major life events					
	During the three months before the mother’s pregnancy		*P* value ^c^	During pregnancy		*P* value ^c^
	Unexposed (N [%])	Exposed (N [%])		Unexposed (N [%])	Exposed (N [%])	
Total ^a^	16,416 (87.96)	2248 (12.04)		16,504 (88.43)	2160 (11.57)	
Mothers						
Age at delivery (years)						
As continuous variable ^b^	29.74 (4.85)	27.94 (4.30)	< 0.001	29.73 (4.83)	27.90 (4.47)	< 0.001
< 25	2155 (13.13)	418 (18.59)	< 0.001	2114 (12.81)	459 (21.25)	< 0.001
25–29	6334 (38.58)	1132 (50.36)		6456 (39.12)	1010 (46.76)	
30–34	4838 (29.47)	469 (20.86)		4847 (29.37)	460 (21.30)	
35–39	2409 (14.67)	169 (7.52)		2406 (14.58)	172 (7.96)	
≥ 40	497 (3.03)	27 (1.20)		500 (3.03)	24 (1.11)	
Missing	183 (1.11)	33 (1.47)		181 (1.10)	35 (1.62)	
Educational level						
Senior high school or below	6214 (37.85)	776 (34.52)	0.002	6215 (37.66)	775 (35.88)	0.114
Undergraduate or above	10,202 (62.15)	1472 (65.48)		10,289 (62.34)	1385 (64.12)	
Occupation						
Professionals	3630 (22.11)	481 (21.40)	0.259	3680 (22.30)	431 (19.95)	0.057
Administrative and services industries	5471 (33.33)	802 (35.68)		5510 (33.39)	763 (35.32)	
Freelancers	241 (1.47)	30 (1.33)		245 (1.48)	26 (1.20)	
Stay-at-home mothers	3832 (23.34)	521 (23.18)		3860 (23.39)	493 (22.82)	
Others	3223 (19.63)	413 (18.37)		3190 (19.33)	446 (20.65)	
Missing	19 (0.12)	1 (0.04)		19 (0.12)	1 (0.05)	
Diseases during the three months before pregnancy						
No	16,098 (98.06)	2188 (97.33)	0.026	16,204 (98.18)	2082 (96.39)	< 0.001
Yes	318 (1.94)	60 (2.67)		300 (1.82)	78 (3.61)	
Medication use during the three months before pregnancy						
No	9235 (56.26)	1156 (51.42)	< 0.001	9203 (55.76)	1188 (55.00)	0.517
Yes	7181 (43.74)	1092 (48.58)		7301 (44.24)	972 (45.00)	
Mode of conception						
Natural conception	15,094 (91.95)	2065 (91.86)	0.919	15,187 (92.02)	1972 (91.30)	0.263
Assisted reproductive technology	1322 (8.05)	183 (8.14)		1317 (7.98)	188 (8.70)	
Parity						
1	6751 (41.12)	1452 (64.59)	< 0.001	6822 (41.34)	1381 (63.94)	< 0.001
2	6607 (40.25)	528 (23.49)		6620 (40.11)	515 (23.84)	
3 or more	934 (5.69)	59 (2.62)		930 (5.63)	63 (2.92)	
Missing	2124 (12.94)	209 (9.30)		2132 (12.92)	201 (9.31)	
Fathers						
Age at child’s birth (years)						
As continuous variable ^b^	32.07 (5.63)	30.11 (4.96)	< 0.001	32.06 (5.61)	30.09 (5.13)	< 0.001
< 25	954 (5.81)	194 (8.63)	< 0.001	933 (5.65)	215 (9.95)	< 0.001
25–29	4948 (30.14)	949 (42.22)		4984 (30.20)	913 (42.27)	
30–34	5265 (32.07)	688 (30.60)		5340 (32.36)	613 (28.38)	
35–39	3204 (19.52)	267 (11.88)		3195 (19.36)	276 (12.78)	
≥ 40	1747 (10.64)	100 (4.45)		1743 (10.56)	104 (4.81)	
Missing	298 (1.82)	50 (2.22)		309 (1.87)	39 (1.81)	
Educational level						
Senior high school or below	6025 (36.70)	817 (36.34)	0.758	5999 (36.35)	843 (39.03)	0.016
Undergraduate or above	10,391 (63.30)	1431 (63.66)		10,505 (63.65)	1317 (60.97)	
Occupation						
Professionals	3840 (23.39)	498 (22.15)	0.057	3882 (23.52)	456 (21.11)	0.015
Administrative and services industries	6186 (37.68)	887 (39.46)		6221 (37.69)	852 (39.44)	
Freelancers	989 (6.02)	149 (6.63)		992 (6.01)	146 (6.76)	
Others	5368 (32.70)	714 (31.76)		5376 (32.57)	706 (32.69)	
Missing	33 (0.20)	0 (0.00)		33 (0.20)	0 (0.00)	
Diseases during the three months before the mother’s pregnancy						
No	16,346 (99.57)	2219 (98.71)	< 0.001	16,402 (99.38)	2126 (98.43)	< 0.001
Yes	70 (0.43)	29 (1.29)		102 (0.62)	34 (1.57)	
Medication use during the three months before the mother’s pregnancy						
No	16,312 (99.37)	2216 (98.58)	< 0.001	16,438 (99.60)	2127 (98.47)	< 0.001
Yes	104 (0.63)	32 (1.42)		66 (0.40)	33 (1.53)	
Children						
Sex						
Boys	8835 (53.82)	1164 (51.78)	0.072	8854 (53.65)	1145 (53.01)	0.592
Girls	7581 (46.18)	1084 (48.22)		7650 (46.35)	1015 (46.99)	

^a^ Row percentage

^b^ Mean (standard deviation)

^c^ Continuous variables were compared using the student’s t-test or Mann–Whitney U test; categorical variables were compared using the χ^2^ test or Fisher’s exact test

**Table 2:** Characteristics of the study population according to maternal exposure to major life events.

Characteristics	Maternal exposure to major life events					
	During the year before pregnancy		*P* value ^c^	During pregnancy		*P* value ^c^
	Unexposed (N [%])	Exposed (N [%])		Unexposed (N [%])	Exposed (N [%])	
Total ^a^	15,653 (83.87)	3011 (16.13)		16,543 (88.64)	2121 (11.36)	
Mothers						
Age at delivery (years)						
As continuous variable ^b^	29.83 (4.89)	27.92 (4.10)	< 0.001	29.75 (4.82)	27.74 (4.50)	< 0.001
< 25	2033 (12.99)	540 (17.93)	< 0.001	2094 (12.66)	479 (22.58)	< 0.001
25–29	5885 (37.60)	1581 (52.51)		6451 (39.00)	1015 (47.85)	
30–34	4694 (29.99)	613 (20.36)		4897 (29.60)	410 (19.33)	
35–39	2367 (15.12)	211 (7.01)		2417 (14.61)	161 (7.59)	
≥ 40	492 (3.14)	32 (1.06)		495 (2.99)	29 (1.37)	
Missing	182 (1.16)	34 (1.13)		189 (1.14)	27 (1.27)	
Educational level						
Senior high school or below	6056 (38.69)	934 (31.02)	< 0.001	6219 (37.59)	771 (36.35)	0.276
Undergraduate or above	9597 (61.31)	2077 (68.98)		10,324 (62.41)	1350 (63.65)	
Occupation						
Professionals	3471 (22.17)	640 (21.26)	< 0.001	3709 (22.42)	402 (18.95)	< 0.001
Administrative and services industries	5147 (32.88)	1126 (37.40)		5495 (33.22)	778 (36.68)	
Freelancers	238 (1.52)	33 (1.10)		247 (1.49)	24 (1.13)	
Stay-at-home mothers	3699 (23.63)	654 (21.72)		3885 (23.48)	468 (22.07)	
Others	3078 (19.66)	558 (18.53)		3187 (19.26)	449 (21.17)	
Missing	20 (0.13)	0 (0.00)		20 (0.12)	0 (0.00)	
Diseases during the three months before pregnancy						
No	15,369 (98.19)	2917 (96.88)	< 0.001	16,248 (98.22)	2038 (96.09)	< 0.001
Yes	284 (1.81)	94 (3.12)		295 (1.78)	83 (3.91)	
Medication use during the three months before pregnancy						
No	8990 (57.43)	1401 (46.53)	< 0.001	9238 (55.84)	1153 (54.36)	0.204
Yes	6663 (42.57)	1610 (53.47)		7305 (44.16)	968 (45.64)	
Mode of conception						
Natural conception	14,357 (91.72)	2802 (93.06)	0.015	15,183 (91.78)	1976 (93.16)	0.031
Assisted reproductive technology	1296 (8.28)	209 (6.94)		1360 (8.22)	145 (6.84)	
Parity						
1	6104 (39.00)	2099 (69.71)	< 0.001	6830 (41.29)	1373 (64.73)	< 0.001
2	6511 (41.60)	624 (20.72)		6625 (40.05)	510 (24.05)	
3 or more	925 (5.91)	68 (2.26)		944 (5.71)	49 (2.31)	
Missing	2113 (13.50)	220 (7.31)		2144 (12.96)	189 (8.91)	
Fathers						
Age at child’s birth (years)						
As continuous variable ^b^	32.17 (5.67)	30.07 (4.81)	< 0.001	32.06 (5.60)	30.08 (5.24)	< 0.001
< 25	915 (5.85)	233 (7.74)	< 0.001	921 (5.57)	227 (10.70)	< 0.001
25–29	4550 (29.07)	1347 (44.74)		5021 (30.35)	876 (41.30)	
30–34	5052 (32.27)	901 (29.92)		5348 (32.33)	605 (28.52)	
35–39	3121 (19.94)	350 (11.62)		3206 (19.38)	265 (12.49)	
≥ 40	1717 (10.97)	130 (4.32)		1736 (10.49)	111 (5.23)	
Missing	298 (1.90)	50 (1.66)		311 (1.88)	37 (1.74)	
Educational level						
Senior high school or below	5846 (37.35)	996 (33.08)	< 0.001	6001 (36.28)	841 (39.65)	0.003
Undergraduate or above	9807 (62.65)	2015 (66.92)		10,542 (63.72)	1280 (60.35)	
Occupation						
Professionals	3637 (23.24)	701 (23.28)	< 0.001	3911 (23.64)	427 (20.13)	< 0.001
Administrative and services industries	5846 (37.35)	1227 (40.75)		6195 (37.45)	878 (41.40)	
Freelancers	941 (6.01)	197 (6.54)		992 (6.00)	146 (6.88)	
Others	5198 (33.21)	884 (29.36)		5413 (32.72)	669 (31.54)	
Missing	31 (0.20)	2 (0.07)		32 (0.19)	1 (0.05)	
Diseases during the three months before the mother’s pregnancy						
No	15,584 (99.56)	2981 (99.00)	< 0.001	16,473 (99.58)	2092 (98.63)	< 0.001
Yes	69 (0.44)	30 (1.00)		70 (0.42)	29 (1.37)	
Medication use during the three months before the mother’s pregnancy						
No	15,559 (99.40)	2969 (98.61)	< 0.001	16,446 (99.41)	2082 (98.16)	< 0.001
Yes	94 (0.60)	42 (1.39)		97 (0.59)	39 (1.84)	
Children						
Sex						
Boys	8453 (54.00)	1546 (51.35)	0.008	8885 (53.71)	1114 (52.52)	0.313
Girls	7200 (46.00)	1465 (48.65)		7658 (46.29)	1007 (47.48)	

^a^ Row percentage

^b^ Mean (standard deviation)

^c^ Continuous variables were compared using the student’s t-test or Mann–Whitney U test; categorical variables were compared using the χ^2^ test or Fisher’s exact test

Compared with children whose fathers experienced no major life events before the mother’s pregnancy, those whose fathers experienced any major life events were at an increased risk of autistic behaviors (adjusted OR after multiple imputations [same below]: 1.33, 95% CI 1.14, 1.55). When considering these events with their inherent nature, paternal experiences of neutral (OR: 1.61, 95% CI 1.29, 2.00) or negative (OR: 1.83, 95% CI 1.30, 2.58) life events before the mother’s pregnancy were associated with an even higher risk of autistic behaviors. Particularly, children of fathers experienced demotion (OR: 2.25, 95% CI 1.40, 3.64), divorce (OR: 3.69, 95% CI 1.33, 10.27) and other major life events (OR: 1.73, 95% CI 1.34, 2.22) showed higher risk of autistic behaviors at preschool age. However, paternal exposure to positive life events before the mother’s pregnancy did not confer a significant difference in the risk of autistic behaviors. Similar results were observed for paternal major life events during the mother’s pregnancy (Table 3). An elevated risk of autistic behaviors was observed in children whose mother was exposed to other types of major life events during pregnancy (OR: 1.90, 95% CI 1.11, 3.26). Maternal exposure to any major life event (crude OR: 1.15, 95% CI 1.00, 1.33), demotion (crude OR: 1.48, 95% CI 1.02, 2.08) and negative events (adjusted OR before multiple imputations: 1.42, 95% CI 1.07, 1.85) before pregnancy were also associated with higher risk of autistic behaviors in preschool children. However, these associations were not observed with multivariable adjustment after multiple imputations (Table 4).

**Table 3:** Association between paternal exposure to major life events and autistic behaviors of preschool children.

Paternal exposure to major life events	Total (N [%])	Autistic behaviors (No. of cases [rate ^a^])	OR (95%CI) ^b^	OR (95%CI) ^c^	OR (95%CI) ^d^
During the three months before the mother’s pregnancy					
Any events					
Unexposed	16,416 (87.96)	1196 (72.86)	Ref	Ref	Ref
Exposed	2248 (12.04)	226 (100.53)	1.42 (1.22, 1.65)	1.40 (1.19, 1.65)	1.33 (1.14, 1.55)
Marriage					
Unexposed	17,592 (94.26)	1327 (75.43)	Ref	Ref	Ref
Exposed	1072 (5.74)	95 (88.62)	1.19 (0.95, 1.47)	1.13 (0.89, 1.42)	1.08 (0.86, 1.35)
Promotion					
Unexposed	18,436 (98.78)	1407 (76.32)	Ref	Ref	Ref
Exposed	228 (1.22)	15 (65.79)	0.85 (0.48, 1.39)	0.95 (0.53, 1.60)	0.86 (0.50, 1.46)
Residential relocation					
Unexposed	18,399 (98.58)	1396 (75.87)	Ref	Ref	Ref
Exposed	265 (1.42)	26 (98.11)	1.32 (0.86, 1.95)	1.49 (0.94, 2.24)	1.35 (0.89, 2.04)
Demotion					
Unexposed	18,537 (99.32)	1401 (75.58)	Ref	Ref	Ref
Exposed	127 (0.68)	21 (165.35)	2.42 (1.47, 3.80)	2.27 (1.30, 3.75)	2.25 (1.40, 3.64)
Divorce					
Unexposed	18,643 (99.89)	1417 (76.01)	Ref	Ref	Ref
Exposed	21 (0.11)	5 (238.10)	3.80 (1.24, 9.71)	3.43 (0.76, 11.49)	3.69 (1.33, 10.27)
Severe illness or death of family members or close friends					
Unexposed	18,504 (99.14)	1406 (75.98)	Ref	Ref	Ref
Exposed	160 (0.86)	16 (100.00)	1.35 (0.77, 2.20)	1.64 (0.93, 2.70)	1.31 (0.77, 2.21)
Others					
Unexposed	18,087 (96.91)	1347 (74.47)	Ref	Ref	Ref
Exposed	577 (3.09)	75 (129.98)	1.86 (1.44, 2.37)	1.77 (1.32, 2.32)	1.73 (1.34, 2.22)
Positive events^e^					
Unexposed	17,406 (93.26)	1317 (75.66)	Ref	Ref	Ref
Exposed	1258 (6.74)	105 (83.47)	1.11 (0.90, 1.36)	1.09 (0.87, 1.35)	1.02 (0.82, 1.26)
Neutral events^e^					
Unexposed	17,823 (95.49)	1322 (74.17)	Ref	Ref	Ref
Exposed	841 (4.51)	100 (118.91)	1.68 (1.35, 2.08)	1.68 (1.31, 2.12)	1.61 (1.29, 2.00)
Negative events^e^					
Unexposed	18,366 (98.40)	1382 (75.25)	Ref	Ref	Ref
Exposed	298 (1.60)	40 (134.23)	1.91 (1.34, 2.64)	1.96 (1.33, 2.80)	1.83 (1.30, 2.58)
During the mother’s pregnancy					
Any events					
Unexposed	16,504 (88.43)	1214 (73.56)	Ref	Ref	Ref
Exposed	2160 (11.57)	208 (96.30)	1.34 (1.15, 1.56)	1.28 (1.07, 1.51)	1.24 (1.06, 1.45)
Marriage					
Unexposed	17,873 (95.76)	1348 (75.42)	Ref	Ref	Ref
Exposed	791 (4.24)	74 (93.55)	1.27 (0.98, 1.61)	1.11 (0.84, 1.44)	1.06 (0.82, 1.36)
Promotion					
Unexposed	18,391 (98.54)	1406 (76.45)	Ref	Ref	Ref
Exposed	273 (1.46)	16 (58.61)	0.75 (0.43, 1.21)	0.84 (0.47, 1.38)	0.77 (0.46, 1.29)
Residential relocation					
Unexposed	18,358 (98.36)	1397 (76.10)	Ref	Ref	Ref
Exposed	306 (1.64)	25 (81.70)	1.08 (0.70, 1.60)	1.32 (0.85, 1.97)	1.12 (0.74, 1.70)
Demotion					
Unexposed	18,127 (97.12)	1361 (75.08)	Ref	Ref	Ref
Exposed	537 (2.88)	61 (113.59)	2.19 (1.42, 3.25)	2.19 (1.36, 3.39)	2.11 (1.38, 3.22)
Divorce					
Unexposed	18,486 (99.05)	1395 (75.46)	Ref	Ref	Ref
Exposed	178 (0.95)	27 (151.69)	7.60 (2.29, 22.81)	11.56 (2.62, 50.90)	7.31 (2.29, 23.27)
Severe illness or death of family members or close friends					
Unexposed	18,651 (99.93)	1417 (75.97)	Ref	Ref	Ref
Exposed	13 (0.07)	5 (384.62)	1.46 (0.98, 2.09)	1.39 (0.90, 2.06)	1.41 (0.96, 2.06)
Others					
Unexposed	18,374 (98.45)	1391 (75.70)	Ref	Ref	Ref
Exposed	290 (1.55)	31 (106.90)	1.58 (1.19, 2.06)	1.44 (1.04, 1.95)	1.46 (1.11, 1.92)
Positive events^e^					
Unexposed	17,640 (94.51)	1336 (75.74)	Ref	Ref	Ref
Exposed	1024 (5.49)	86 (83.98)	1.12 (0.89, 1.40)	1.04 (0.81, 1.33)	0.98 (0.77, 1.24)
Neutral events^e^					
Unexposed	17,825 (95.50)	1336 (74.95)	Ref	Ref	Ref
Exposed	839 (4.50)	86 (102.50)	1.41 (1.11, 1.76)	1.42 (1.09, 1.82)	1.36 (1.07, 1.71)
Negative events^e^					
Unexposed	18,195 (97.49)	1362 (74.86)	Ref	Ref	Ref
Exposed	469 (2.51)	60 (127.93)	1.81 (1.36, 2.37)	1.75 (1.27, 2.36)	1.76 (1.33, 2.33)

OR = Odds Ratio, CI = Confidence Interval

^a^ Rate was calculated as the number of preschool children screened positive for autistic behaviors per thousand children in each stratum

^b^ Unadjusted model

^c^ For paternal major life events before the mother’s pregnancy, models were adjusted for maternal educational level, age at the child’s birth, occupations and mode of conception, paternal educational level, age at the child’s birth, occupations, and child’s sex. For paternal major life events during the mother’s pregnancy, models were further adjusted for parental diseases and medication use before the mother’s pregnancy

^d^ Adjusted models after multiple imputations

**Table 4:** Association between maternal exposure to major life events and autistic behaviors of preschool children.

Maternal exposure to major life events	Total (N [%])	Autistic behaviors (No. of cases [rate ^a^])	OR (95%CI) ^b^	OR (95%CI) ^c^	OR (95%CI) ^d^
During the year before pregnancy					
Any events					
Unexposed	15,653 (83.87)	1166 (74.49)	Ref	Ref	Ref
Exposed	3011 (16.13)	256 (85.02)	1.15 (1.00, 1.33)	1.13 (0.96, 1.32)	1.09 (0.94, 1.26)
Marriage					
Unexposed	16,728 (89.63)	1261 (75.38)	Ref	Ref	Ref
Exposed	1936 (10.37)	161 (83.16)	1.11 (0.93, 1.32)	1.03 (0.85, 1.24)	1.02 (0.85, 1.22)
Promotion					
Unexposed	18,419 (98.69)	1404 (76.23)	Ref	Ref	Ref
Exposed	245 (1.31)	18 (73.47)	0.96 (0.57, 1.51)	0.83 (0.45, 1.41)	0.99 (0.61, 1.61)
Residential relocation					
Unexposed	18,139 (97.19)	1379 (76.02)	Ref	Ref	Ref
Exposed	525 (2.81)	43 (81.90)	1.08 (0.78, 1.47)	1.05 (0.73, 1.46)	1.10 (0.80, 1.52)
Demotion					
Unexposed	18,340 (98.26)	1387 (75.63)	Ref	Ref	Ref
Exposed	324 (1.74)	35 (108.02)	1.48 (1.02, 2.08)	1.45 (0.97, 2.09)	1.40 (0.97, 2.00)
Divorce					
Unexposed	18,642 (99.88)	1420 (76.17)	Ref	Ref	Ref
Exposed	22 (0.12)	2 (90.91)	1.21 (0.19, 4.16)	1.51 (0.23, 5.67)	0.97 (0.22, 4.21)
Severe illness or death of family members or close friends					
Unexposed	18,295 (98.02)	1390 (75.98)	Ref	Ref	Ref
Exposed	369 (1.98)	32 (86.72)	1.15 (0.79, 1.64)	1.33 (0.89, 1.93)	1.17 (0.81, 1.69)
Others					
Unexposed	18,567 (99.48)	1411 (76.00)	Ref	Ref	Ref
Exposed	97 (0.52)	11 (113.40)	1.56 (0.78, 2.79)	1.36 (0.60, 2.68)	1.37 (0.72, 2.59)
Positive events^e^					
Unexposed	16,548 (88.66)	1247 (75.36)	Ref	Ref	Ref
Exposed	2116 (11.34)	175 (82.70)	1.11 (0.94, 1.30)	1.02 (0.84, 1.22)	1.02 (0.86, 1.21)
Neutral events^e^					
Unexposed	18,045 (96.68)	1368 (75.81)	Ref	Ref	Ref
Exposed	619 (3.32)	54 (87.24)	1.17 (0.87, 1.53)	1.10 (0.79, 1.49)	1.16 (0.87, 1.54)
Negative events^e^					
Unexposed	17,969 (96.28)	1355 (75.41)	Ref	Ref	Ref
Exposed	695 (3.72)	67 (96.40)	1.31 (1.00, 1.68)	1.42 (1.07, 1.85)	1.27 (0.98, 1.65)
During pregnancy					
Any events					
Unexposed	16,543 (88.64)	1239 (74.90)	Ref	Ref	Ref
Exposed	2121 (11.36)	183 (86.28)	1.17 (0.99, 1.37)	1.02 (0.85, 1.22)	1.08 (0.91, 1.27)
Marriage					
Unexposed	17,814 (95.45)	1344 (75.45)	Ref	Ref	Ref
Exposed	850 (4.55)	78 (91.76)	1.24 (0.97, 1.56)	1.02 (0.78, 1.32)	1.04 (0.81, 1.33)
Promotion					
Unexposed	18,555 (99.42)	1415 (76.26)	Ref	Ref	Ref
Exposed	109 (0.58)	7 (64.22)	0.83 (0.35, 1.66)	0.66 (0.20, 1.60)	0.90 (0.42, 1.96)
Residential relocation					
Unexposed	18,248 (97.77)	1389 (76.12)	Ref	Ref	Ref
Exposed	416 (2.23)	33 (79.33)	1.05 (0.72, 1.47)	1.12 (0.75, 1.61)	1.00 (0.70, 1.44)
Demotion					
Unexposed	18,280 (97.94)	1384 (75.71)	Ref	Ref	Ref
Exposed	384 (2.06)	38 (98.96)	1.34 (0.94, 1.86)	1.22 (0.83, 1.74)	1.25 (0.89, 1.77)
Divorce					
Unexposed	18,647 (99.91)	1421 (76.21)	Ref	Ref	Ref
Exposed	17 (0.09)	1 (58.82)	0.76 (0.04, 3.71)	1.08 (0.06, 5.83)	0.63 (0.08, 4.83)
Severe illness or death of family members or close friends					
Unexposed	18,164 (97.32)	1386 (76.30)	Ref	Ref	Ref
Exposed	500 (2.68)	36 (72.00)	0.94 (0.65, 1.30)	0.80 (0.52, 1.18)	0.93 (0.66, 1.32)
Others					
Unexposed	18,551 (99.39)	1406 (75.79)	Ref	Ref	Ref
Exposed	113 (0.61)	16 (141.59)	2.01 (1.14, 3.32)	1.06 (0.44, 2.16)	1.90 (1.11, 3.26)
Positive events^e^					
Unexposed	17,721 (94.95)	1338 (75.50)	Ref	Ref	Ref
Exposed	943 (5.05)	84 (89.08)	1.20 (0.94, 1.50)	1.00 (0.77, 1.29)	1.03 (0.81, 1.31)
Neutral events^e^					
Unexposed	18,139 (97.19)	1373 (75.69)	Ref	Ref	Ref
Exposed	525 (2.81)	49 (93.33)	1.26 (0.92, 1.68)	1.12 (0.78, 1.56)	1.20 (0.89, 1.63)
Negative events^e^					
Unexposed	17,779 (95.26)	1350 (75.93)	Ref	Ref	Ref
Exposed	885 (4.74)	72 (81.36)	1.08 (0.83, 1.37)	0.98 (0.74, 1.29)	1.04 (0.81, 1.34)

OR = Odds Ratio, CI = Confidence Interval

^a^ Rate was calculated as the number of preschool children screened positive for autistic behaviors per thousand children in each stratum

^b^ Unadjusted model

^c^ For maternal major life events before pregnancy, models were adjusted for maternal educational level, age at the child’s birth, occupations and mode of conception, paternal educational level, age at the child’s birth, occupations, and child’s sex. For maternal major life events during pregnancy, models were further adjusted for parental diseases and medication use before the mother’s pregnancy

^d^ Adjusted models after multiple imputations

^e^ Positive events included marriage and promotion; neutral events included residential relocation, other life events; negative events included demotion, divorce and severe illness or death of family members or friends

When examining the joint effect of parental exposure to major life events before pregnancy on autistic behaviors in preschool children, both dual parental exposure (OR: 1.29, 95% CI 1.07, 1.56) and paternal-only exposure (OR: 1.38, 95% CI 1.09, 1.75) were associated with an increased risk of autistic behaviors. During pregnancy, paternal-only exposure but not dual exposure was associated with higher risk (OR: 1.28, 95% CI 1.02, 1.60) of autistic behaviors. No associations were observed when only the mother experienced major life events (Supplementary Table S2).

When examining the cumulative number of parental major life events, we observed a dose–response relationship for paternal exposure. Compared with children whose fathers experienced no major life events during the three months before pregnancy, those whose fathers experienced one event showed higher risk of autistic behaviors (OR: 1.30, 95% CI 1.11, 1.53), and the risk was even higher when fathers experienced two or more events (OR: 1.70, 95% CI 1.08, 2.68). A similar pattern was observed for paternal exposure during pregnancy, with elevated risks for both one event (OR: 1.19, 95% CI 1.00, 1.40) and two or more events (OR: 1.79, 95% CI 1.17, 2.73). No significant associations were found for maternal exposure, regardless of the number or timing of exposure (Supplementary Table S3).

When fathers experienced major life events before or during pregnancy, both parents were more inclined to consume alcohol and smoke during these periods (Supplementary Table S4). Mothers who were exposed to major life events before pregnancy were also more likely to consume alcohol before pregnancy, whereas such exposure during pregnancy was linked to a higher proportion of both alcohol consumption and smoking. Furthermore, when mothers experienced major life events before or during pregnancy, fathers were also more prone to consume alcohol and smoke (Supplementary Table S5). The rate of autistic behaviors among children was higher when parents both experienced major life events and consumed alcohol (Supplementary Tables S6 and S7).

**Fig. 1:**
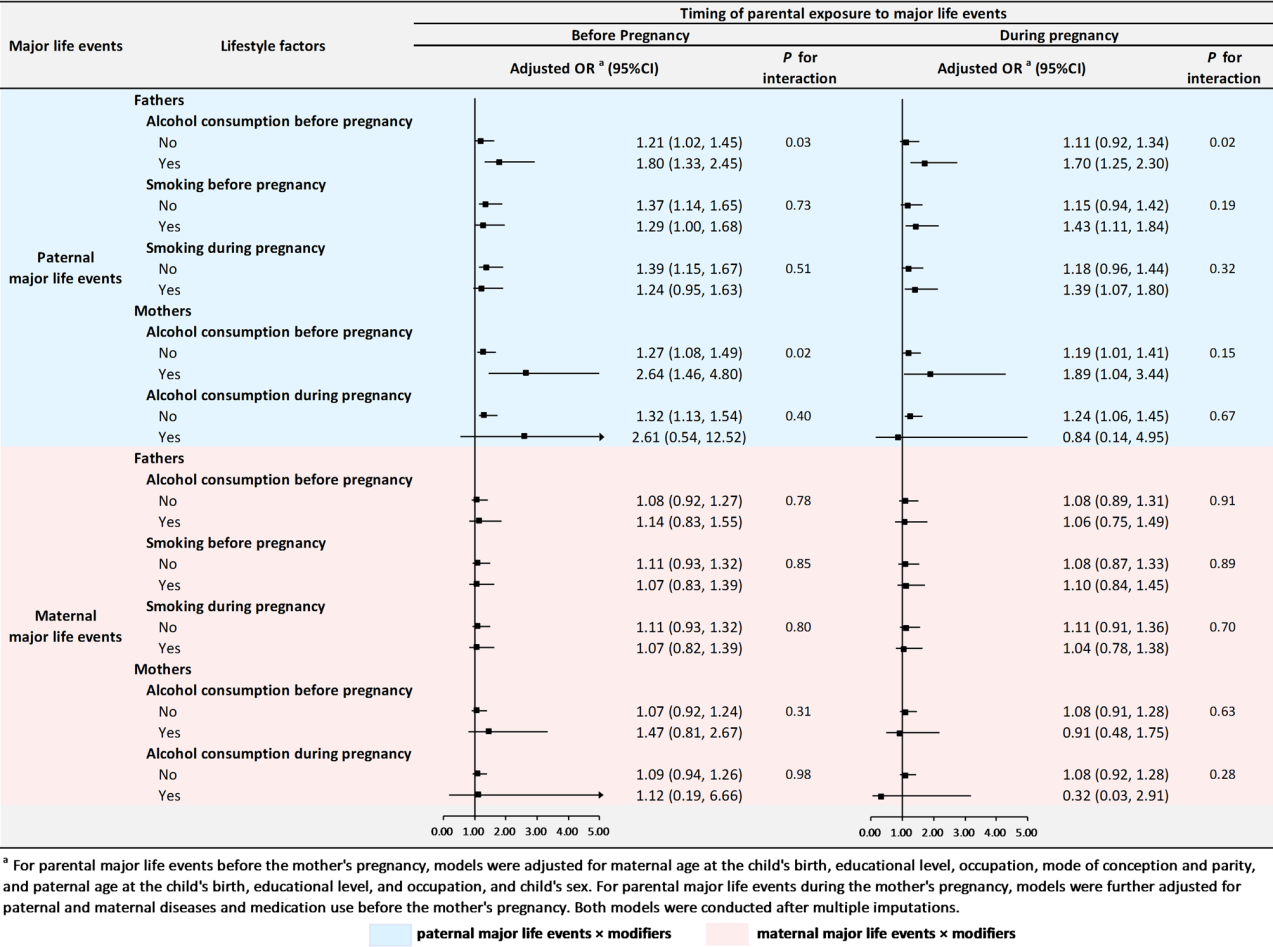
Interaction effect between parental major life events and alcohol consumption or smoking on autistic behaviors in preschool children.

Among children whose fathers did not consume alcohol before pregnancy, paternal experience of major life events before pregnancy was associated with a greater risk of autistic behaviors (OR: 1.21, 95% CI 1.02, 1.45); such association was stronger in children whose fathers consumed alcohol before pregnancy (OR:1.80, 95% CI 1.33, 2.45; *P* for interaction: 0.03). Similarly, such association was also intensified among children whose mothers consumed alcohol before pregnancy (OR: 2.64, 95% CI 1.46, 4.80) than among those without such exposure (OR: 1.27, 95% CI 1.08, 1.49; *P* for interaction: 0.02). Paternal alcohol consumption before pregnancy also modified the association between paternal major life events during pregnancy and autistic behaviors (*P* for interaction: 0.02). No evidence of a modifying effect was observed for parental smoking (Fig. 1 and Supplementary Figure S3).

When stratified by the sex of the child, the overall association of parental major life events before or during pregnancy with the risk of autistic behaviors remained similar among boys. However, only paternal exposure to major life events before the mother’s pregnancy was associated with a higher risk of autistic behaviors among girls. Nevertheless, we did not observe any difference in the association between parental major life events and autistic behaviors between the two sexes (all *P* for interaction > 0.05) (Supplementary Table S8).

## Discussion

This study found that major life events experienced by fathers before or during the mother’s pregnancy, especially neutral or negative ones, were associated with a higher risk of autistic behaviors in preschoolers. Maternal exposure to other types of major life events during pregnancy was also associated with a higher risk of autistic behaviors. Pre-pregnancy alcohol consumption by either parent exacerbated the impact of major life events experienced by fathers before the mother’s pregnancy on the risk of autistic behaviors among preschool children.

Major life events are important sources of stress from social and psychological factors, which have a direct or indirect impact on an individual’s mental health. Few epidemiologic studies have reported the association between paternal experience of major life events and autistic behaviors in offspring. However, fathers’ exposure to major life events that caused stress plays a significant role in the development and well-being of their children^20^. Previous studies have demonstrated the association of paternal stress before the mother’s pregnancy and neurodevelopmental outcome of children, which aligns with our findings^21^. Paternal stress before pregnancy may affect fetal programming and subsequently lead to neurodevelopmental abnormalities in offspring. This can occur through mechanisms such as affecting sperm quality via DNA methylation, small non-coding RNAs, and histone post-translational modifications^20^.

The current study also showed an association between paternal exposure to major life events during the mother’s pregnancy and autistic behaviors in children, supporting evidence from previous observations^22^. This suggests other possible pathways through which paternal major life events affect the neurodevelopment of the child, including influencing paternal direct caregiving to children, transmitting stress between parents within the family environment, and impacting mother–child interactions. The stress experienced by a husband can significantly impact the stress levels of his wife, especially during major life events such as marital conflicts or financial difficulties. Fathers’ stress also influences maternal well-being and mother-infant bonding which affect children’s development. This may explain why the impact of paternal major life events was mainly driven by negative events, including demotion and divorce^8,23,24^. Future work is needed to unravel how paternal major life events affect the development of autistic behavior in offspring through influencing the health of both parents, parenting practices, and overall family dynamics.

Unlike paternal major life events, there has been a significant focus on maternal major life events and children’s developmental outcomes. The current study found that maternal exposure to other major life events during pregnancy, such as marital discord, family conflicts, and experiences of deception or financial fraud, was associated with an increased risk of autistic behaviors in preschool-aged children. This is consistent with previous studies linking maternal exposure to stressful life events such as financial hardship, interpersonal conflicts, and sleep disruptions during pregnancy to a higher risk of ASD or autistic behaviors in offspring^5,7,25,26^. However, it is important to note that some other studies have not established such associations^27,28^. We did not find significant associations between maternal major life events before pregnancy and autistic behaviors after adjustment for confounders, which was aligned with some previous studies^27–29^, but in contrast to others^30,31^. This inconsistency may be attributed to variations in study design, types and measurements of major life events, as well as differences in genetic and socio-economic backgrounds of study populations. Maternal pre-pregnancy and prenatal exposure to major life events may influence children’s development through various mechanisms. For example, maternal stress exposure can lead to the release of catecholamine, reducing blood flow to the uterus and placenta. This impairs hormone transportation and causes fetal hypoxia, increasing the risk of ASD in children^32^. In addition, stress from major life events may trigger the release of hormones that disrupt the development of the fetal hypothalamic–pituitary–adrenal (HPA) axis^33^, and induce long-term epigenetic modifications that may increase the subsequent risk of autistic behaviors^10,34^. Further research should prioritize investigations of stress-induced alterations in parental germline cells and offspring’s DNA^35,36^.

Our study showed that alcohol consumption of both parents before the mother’s pregnancy strengthened the association between paternal major life events before pregnancy and autistic behaviors in children. This finding aligns with evidence from animal studies, which have demonstrated that paternal alcohol exposure before conception can alter levels of key neurodevelopmental factors in offspring, such as brain nerve growth factor and brain-derived neurotrophic factor^37^. In addition, alcohol may induce alterations in the epigenetic landscape of sperm cells, leading to changes in DNA methylation patterns, histone modifications, and RNA content during spermatogenesis, which may have adverse effects on fetal development^38^. Maternal pre-pregnancy alcohol consumption may activate the HPA axis^39^, resulting in altered stress responses, dysregulated stress hormone levels, and increased vulnerability to stress-related disorders in offspring. These mechanisms are similar to the potential pathways linking major life events and ASD, suggesting that major life events and alcohol consumption might influence autistic behaviors in children through mutual biological processes.

We did not find evidence for an interaction between parental major life events and paternal smoking before or during pregnancy in relation to autistic behaviors in children. This finding stands despite evidence from animal models indicating that prenatal nicotine exposure may affect offspring neurodevelopment, including sensorimotor gating deficits and cognitive impairments^40^. Furthermore, previous human studies have suggested that paternal smoking may influence offspring neurodevelopment through pathways involving epigenetic alterations or oxidative stress^41^. A key methodological consideration is our classification of both former smokers and non-smokers as “non-smoking” during the specified period. This classification may lead to an underestimation of the true association, as former smokers might also interact with major life events and influence autistic behaviors in children through epigenetic modifications. However, the limited number of former smokers in the study population precluded meaningful stratified analyses of this subgroup. Consequently, our study could not adequately evaluate the potential modifying effect of former smoking status. This limitation underscores the need for future large-scale studies with sufficient power to specifically examine the role of paternal smoking history, particularly former smoking, in modifying the associations between parental major life events and child neurodevelopment. Furthermore, the reliance on self-reported smoking status may have introduced reporting bias, as current or former smokers might be more likely to underreport their smoking behaviors before or during pregnancy^42^. Lastly, it is important to acknowledge that unmeasured confounding factors, such as parental socioeconomic status or mental health, may also have influenced the observed associations.

There are several strengths in this study. First, we had a sufficiently large sample size to support a thorough investigation of the association between parental major life events and autistic behaviors among children. Second, the results showed consistency between crude and adjusted models, lending support to the robustness of the studied associations. Third, in addition to maternal major life events, we also explored the impact of paternal major life events on children’s autistic behaviors, providing novel evidence of paternal contributions to children’s neurodevelopmental outcomes. Fourth, we assessed the modifying effect of alcohol consumption and smoking, providing new insights into the potential pathways through which lifestyle factors may interact with major life events to contribute to the development of autistic behaviors. However, this study also has limitations. First, the retrospectively collected information on parental major life events might be subject to recall bias. Second, our study only included children from one Chinese urban area, which potentially limits the generalizability of the results. Third, we lacked the power to analyze the interaction between parental major life events and maternal smoking status due to the small number of mothers who smoked. Fourth, while we specifically examined exposures to major life events before or during pregnancy, we did not assess parental exposures during puberty, which could represent an additional influential factor for child neurodevelopment. This limitation underscores the need for future longitudinal studies to trace paternal exposures across the life course and their potential epigenetic or behavioral impacts on offspring neurodevelopment. Finally, a key limitation of our study is the lack of data on parental history of neurodevelopmental disorders. This information gap precludes a comprehensive analysis of how genetic factors and major life events before or during pregnancy may jointly contribute to autistic behaviors.

## Conclusions

This study provides new evidence on the association between major life events experienced by parents, particularly fathers, and autistic behaviors in preschool children. The association between pre-pregnancy paternal major life events and the risk of autistic behaviors is notably stronger when combined with parental alcohol consumption before pregnancy. Maternal exposure to major life events during pregnancy is also associated with autistic behaviors, whereas no such association is found for maternal major life events occurring before pregnancy. These findings underscore the necessity for targeted interventions to provide support for the affected parents and to improve neurodevelopmental outcomes for their children.

## Supplementary Information

Below is the link to the electronic supplementary material.

Supplementary Material 1

## Data Availability

The datasets generated and/or analyzed during the current study are not publicly available due to ethical restrictions but are available from the corresponding author on reasonable request.
