# Supplementary material for: Parental major life events before or during pregnancy and autistic behaviors among preschool children
Source: Sci Rep. 2025 Oct 31;15:38217. doi: 10.1038/s41598-025-22094-z (PMC12578796; doi:10.1038/s41598-025-22094-z)
Supplement: Supplementary file 1 — Supplementary Material 1 [file 41598_2025_22094_MOESM1_ESM.docx]

**Supplementary Information**

**Parental major life events before or during pregnancy and autistic behaviors among preschool children**

Weiying Liu, Yulan Wu, Dongyan Wen, Huiting Chen, Jinming Liu, Shuang Wu,

Jiarong Lin, Zizi Liu, Xuanshu Wang, Lvping Li, Feixiang Zeng, Ruoqing Chen, Yu Jin

**Supplementary Methods.** Multiple-choice questions used to assess the major life events of parents.

**Supplementary Figure S1.** Directed Acyclic Graph (DAG) of the hypothesized causal relationships between parental major life events before pregnancy, confounders, and autistic behaviors in preschoolers.

**Supplementary Figure S2.** Directed acyclic graph (DAG) of the hypothesized causal relationships between parental major life events during pregnancy, confounders, and autistic behaviors in preschoolers.

**Supplementary Figure S3.** Interaction effect between parental major life events and paternal smoking (never/former/current) on autistic behaviors in preschool children

**Supplementary Table S1.** Classification of occupations for fathers and mothers

**Supplementary Table S2.** Association between parental joint exposure to major life events and autistic behaviors in preschool children

**Supplementary Table S3.** Association between numbers of parental major life events and autistic behaviors in preschool children

**Supplementary Table S4.** Parental alcohol consumption and smoking according to paternal exposure to major life events

**Supplementary Table S5.** Parental alcohol consumption and smoking according to maternal exposure to major life events

**Supplementary Table S6.** Parental alcohol consumption and smoking and autistic behaviors according to paternal exposure to major life events

**Supplementary Table S7.** Parental alcohol consumption and smoking and autistic behaviors according to maternal exposure to major life events

**Supplementary Table S8.** Association between parental major life events and autistic behaviors in preschool children stratified by the sex of the child

**Supplementary Methods. Multiple-choice questions used to assess the major life events of parents.**

Q38. Did the mother experience any major life events in the year before pregnancy? [Multiple choices]

☐ Marriage

☐ Divorce

☐ Relocation

☐ Job promotion

☐ Job demotion or loss

☐ Severe illness or death of a family member or close friend

☐ Others

☐ None

Q39. Did the mother experience any major life events during pregnancy? [Multiple choices]

☐ Marriage

☐ Divorce

☐ Relocation

☐ Job promotion

☐ Job demotion or loss

☐ Severe illness or death of a family member or close friend

☐ Others

☐ None

Q58. Did the child’s father experience any major life events in the three months before the mother’s pregnancy (while she was not yet pregnant)? [Multiple choices]

☐ Marriage

☐ Divorce

☐ Relocation

☐ Job promotion

☐ Job demotion or loss

☐ Severe illness or death of a family member or close friend

☐ Others

☐ None

Q59. Did the child’s father experience any major life events during the mother’s pregnancy? [Multiple choices]

☐ Marriage

☐ Divorce

☐ Relocation

☐ Job promotion

☐ Job demotion or loss

☐ Severe illness or death of a family member or close friend

☐ Others

☐ None

**Supplementary Figure S1. Directed acyclic graph (DAG) of the hypothesized causal relationships between parental major life events before pregnancy, confounders, and autistic behaviors in preschoolers.**


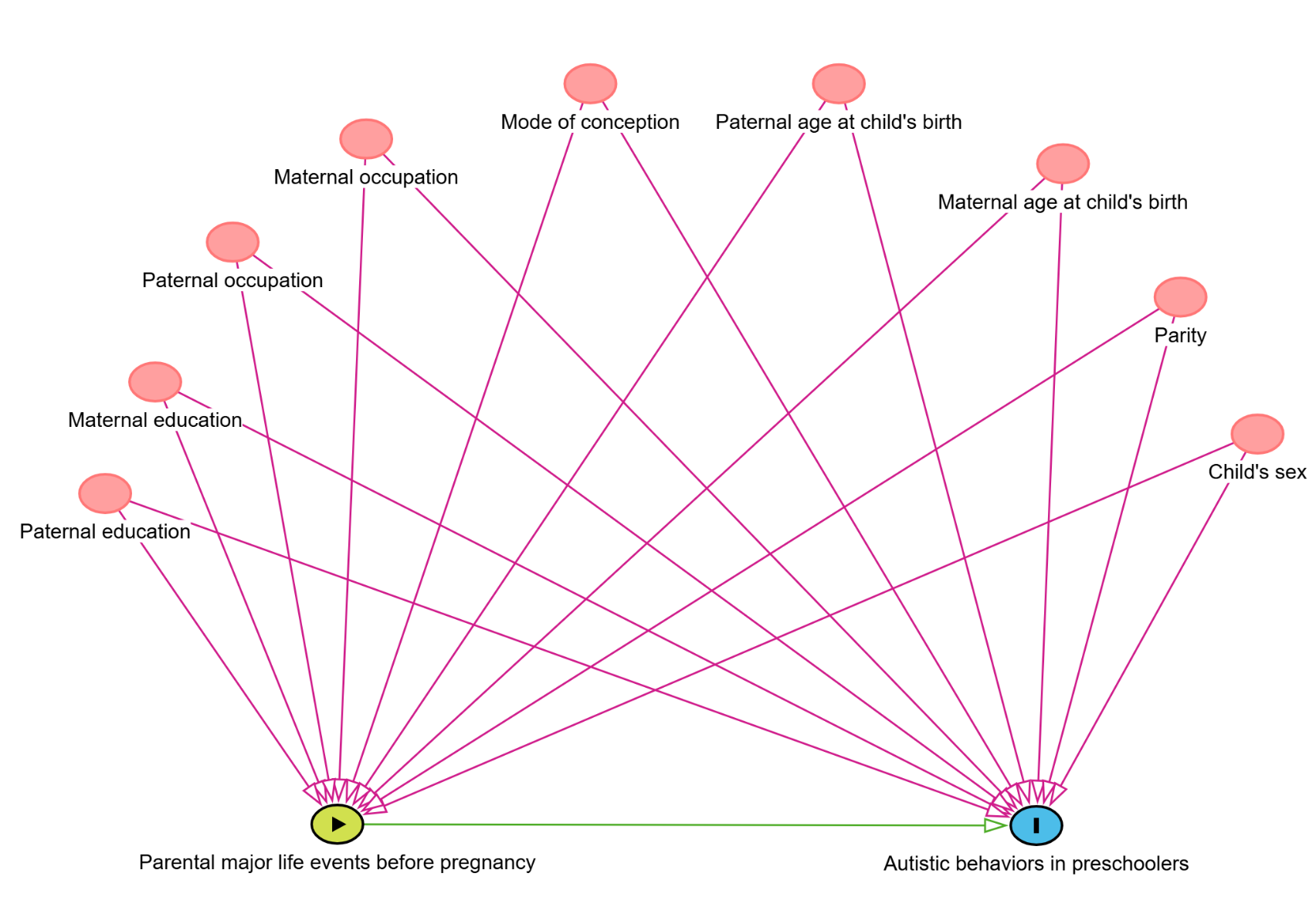


**Supplementary Figure S2. Directed acyclic graph (DAG) of the hypothesized causal relationships between parental major life events during pregnancy, confounders, and autistic behaviors in preschoolers.**


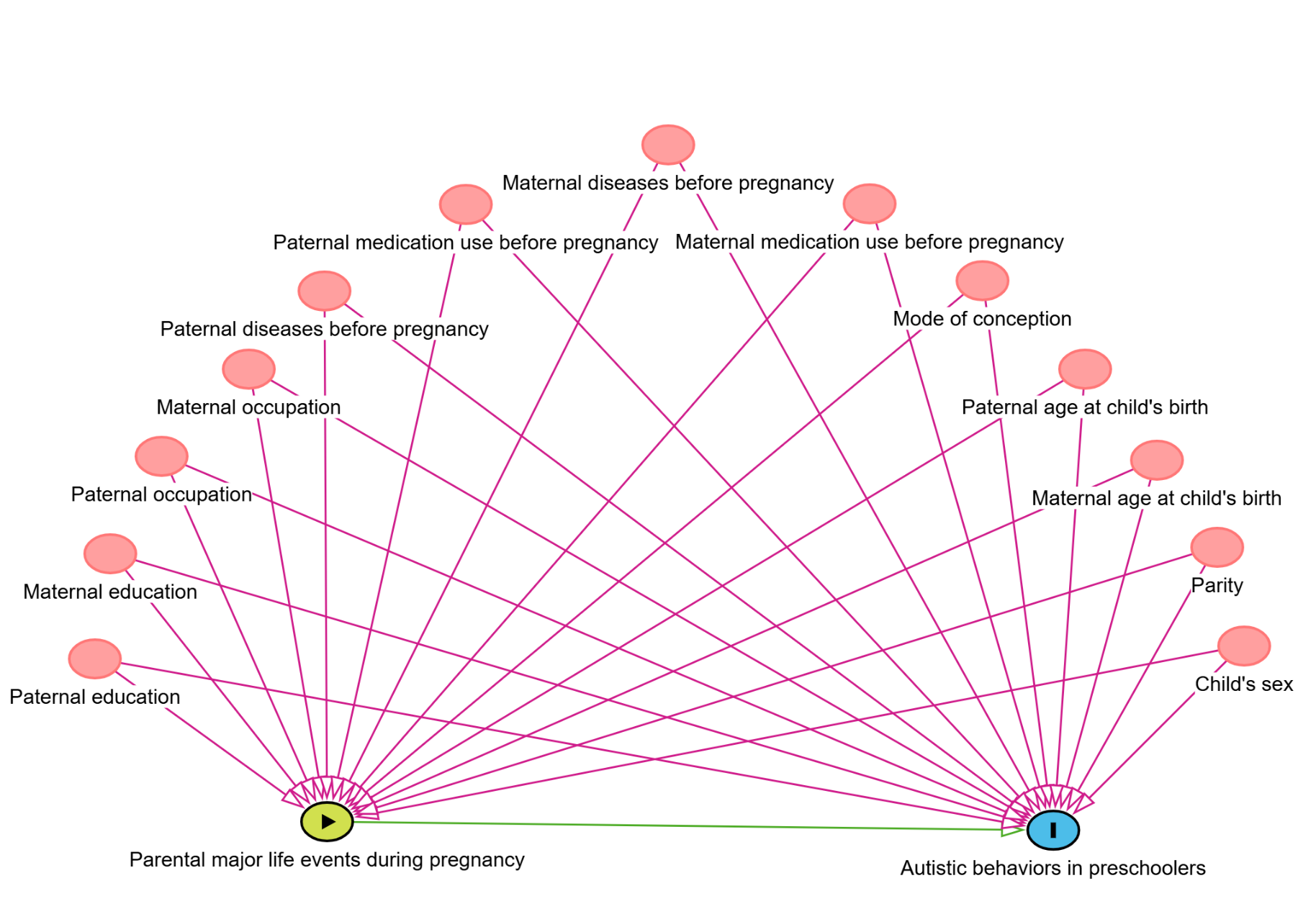


**Supplementary Figure S3. Interaction effect between parental major life events and paternal smoking (never/former/current) on autistic behaviors in preschool children**

| **Supplementary Table S1. Classification of occupations for fathers and mothers** | |
| --- | --- |
| **Categories** | **Occupations** |
| **Fathers** |  |
| Professionals | Government, enterprise, and institutional leaders; Teachers; Medical professionals; Engineers, laboratory technicians, IT professionals, and research technicians |
| Administrative and service workers | Office workers; Business and service industry workers |
| Freelancers | Freelancers |
| Others | Agricultural, forestry, fishery, animal husbandry, and water conservancy workers; Production and transportation equipment operators and related personnel; Military personnel; Stay-at-home parent |
| **Mothers** |  |
| Professional services | Government, enterprise, and institutional leaders; Teachers; Medical professionals; Engineers, laboratory technicians, IT professionals, and research technicians |
| Administrative and service workers | Office workers; Business and service industry workers |
| Freelancers | Freelancers |
| Stay-at-home mothers | Stay-at-home parent |
| Others | Agricultural, forestry, fishery, animal husbandry, and water conservancy workers; Production and transportation equipment operators and related personnel; Military personnel |

| **Supplementary Table S2. Association between parental joint exposure to major life events and autistic behaviors in preschool children** | | | | | |
| --- | --- | --- | --- | --- | --- |
| **Parental joint exposure to major life events** | **Total (N [%])** | **Autistic behaviors**  **(No. of cases [rate ^a^])** | **OR (95%CI) ^b^** | **OR (95%CI) ^c^** | **OR (95%CI) ^d^** |
| **Before pregnancy** |  |  |  |  |  |
| No | 14854 (79.59) | 1082 (72.84) | Ref. | Ref. | Ref. |
| Only father | 799 (4.28) | 84 (105.13) | 1.50 (1.18, 1.88) | 1.42 (1.09, 1.84) | 1.38 (1.09, 1.75) |
| Only mother | 1562 (8.37) | 114 (72.98) | 1.00 (0.82, 1.22) | 0.96 (0.77, 1.19) | 0.96 (0.78, 1.18) |
| Both parents | 1449 (7.76) | 142 (98.00) | 1.38 (1.15, 1.66) | 1.38 (1.12, 1.68) | 1.29 (1.07, 1.56) |
| **During pregnancy** |  |  |  |  |  |
| No | 15577 (83.46) | 1146 (73.57) | Ref. | Ref. | Ref. |
| Only father | 966 (5.18) | 93 (96.27) | 1.34 (1.07, 1.67) | 1.30 (1.01, 1.65) | 1.28 (1.02, 1.60) |
| Only mother | 927 (4.97) | 68 (73.35) | 1.00 (0.77, 1.28) | 0.81 (0.59, 1.07) | 0.96 (0.74, 1.25) |
| Both parents | 1194 (6.4) | 115 (96.31) | 1.34 (1.09, 1.63) | 1.22 (0.97, 1.52) | 1.20 (0.97, 1.47) |
| OR = Odds Ratio, CI = Confidence Interval | | | | | |
| ^a^ Rate was calculated as the number of preschool children screened positive for autistic behaviors per thousand children in each stratum. | | | | | |
| ^b^ Unadjusted model | | | | | |
| ^c^ For parental major life events before the mother's pregnancy, models were adjusted for maternal educational level, age at the child's birth, occupations and mode of conception, paternal educational level, age at the child's birth, occupations, and child's sex. For parental major life events during the mother's pregnancy, models were further adjusted for parental diseases and medication use before the mother's pregnancy. | | | | | |
| ^d^ Adjusted models after multiple imputations. | | | | | |

| **Supplementary Table S3. Association between numbers of parental major life events and autistic behaviors in preschool children.** | | | | | |
| --- | --- | --- | --- | --- | --- |
| **Numbers of major life events** | **Total (N [%])** | **Autistic behaviors**  **(No. of cases [rate ^a^])** | **OR (95%CI) ^b^** | **OR (95%CI) ^c^** | **OR (95%CI) ^d^** |
| **Fathers** |  |  |  |  |  |
| **During the three months before the mother's pregnancy** |  |  |  |  |  |
| 0 | 16416 (87.96) | 1196 (72.86) | Ref. | Ref. | Ref. |
| 1 | 2070 (11.09) | 204 (98.55) | 1.39 (1.19, 1.62) | 1.36 (1.15, 1.62) | 1.30 (1.11, 1.53) |
| ≥2 | 178 (0.95) | 22 (123.60) | 1.79 (1.11, 2.75) | 1.84 (1.11, 2.90) | 1.70 (1.08, 2.68) |
| **During the mother's pregnancy** | |  |  |  |  |
| 0 | 16504 (88.43) | 1214 (73.56) | Ref. | Ref. | Ref. |
| 1 | 1963 (10.52) | 182 (92.72) | 1.29 (1.09, 1.51) | 1.22 (1.01, 1.45) | 1.19 (1.00, 1.40) |
| ≥2 | 197 (1.05) | 26 (131.98) | 1.91 (1.23, 2.85) | 1.94 (1.20, 2.99) | 1.79 (1.17, 2.73) |
| **Mothers** |  |  |  |  |  |
| **During the year before pregnancy** |  |  |  |  |  |
| 0 | 15653 (83.87) | 1166 (74.49) | Ref. | Ref. | Ref. |
| 1 | 2574 (13.79) | 222 (86.25) | 1.17 (1.01, 1.36) | 1.16 (0.98, 1.36) | 1.11 (0.95, 1.30) |
| ≥2 | 437 (2.34) | 34 (77.80) | 1.05 (0.72, 1.47) | 0.95 (0.63, 1.38) | 0.98 (0.68, 1.41) |
| **During pregnancy** |  |  |  |  |  |
| 0 | 16543 (88.64) | 1239 (74.90) | Ref. | Ref. | Ref. |
| 1 | 1892 (10.14) | 160 (84.57) | 1.14 (0.96, 1.35) | 1.01 (0.83, 1.22) | 1.06 (0.89, 1.27) |
| ≥2 | 229 (1.22) | 23 (100.44) | 1.38 (0.87, 2.08) | 1.12 (0.67, 1.78) | 1.20 (0.77, 1.86) |
| OR = Odds Ratio, CI = Confidence Interval | | | | | |
| ^a^ Rate was calculated as the number of preschool children screened positive for autistic behaviors per thousand children in each stratum. | | | | | |
| ^b^ Unadjusted model | | | | | |
| ^c^ For parental major life events before the mother's pregnancy, models were adjusted for maternal educational level, age at the child's birth, occupations and mode of conception, paternal educational level, age at the child's birth, occupations, and child's sex. For parental major life events during the mother's pregnancy, models were further adjusted for parental diseases and medication use before the mother's pregnancy. | | | | | |
| ^d^ Adjusted models after multiple imputations. | | | | | |

| **Supplementary Table S4. Parental alcohol consumption and smoking according to paternal exposure to major life events** | | | | | | |
| --- | --- | --- | --- | --- | --- | --- |
| **Characteristics** | **Paternal exposure to major life events** | | | | | |
|  | **During the three months before the mother's pregnancy** | | | **During the mother's pregnancy** | | |
|  | **Unexposed (N [%])** | **Exposed (N [%])** | ***P* value ^a^** | **Unexposed (N [%])** | **Exposed (N [%])** | ***P* value ^a^** |
| **Total ^b^** | 16416 (87.96) | 2248 (12.04) |  | 16504 (88.43) | 2160 (11.57) |  |
| **Fathers** |  |  |  |  |  |  |
| **Alcohol consumption before pregnancy** |  |  |  |  |  |  |
| No | 13868 (84.48) | 1736 (77.22) | <0.001 | 13981 (84.71) | 1623 (75.14) | <0.001 |
| Yes | 2548 (15.52) | 512 (22.78) |  | 2523 (15.29) | 537 (24.86) |  |
| **Smoking before pregnancy** |  |  |  |  |  |  |
| Never | 11173 (68.06) | 1366 (60.77) | <0.001 | 11297 (68.45) | 1242 (57.50) | <0.001 |
| Former | 448 (2.73) | 79 (3.51) |  | 455 (2.76) | 72 (3.33) |  |
| Current | 4795 (29.21) | 803 (35.72) |  | 4752 (28.79) | 846 (39.17) |  |
| **Smoking during pregnancy** |  |  |  |  |  |  |
| Never | 11577 (70.52) | 1448 (64.41) | <0.001 | 11714 (70.98) | 1311 (60.69) | <0.001 |
| Former | 308 (1.88) | 51 (2.27) |  | 311 (1.88) | 48 (2.22) |  |
| Current | 4531 (27.60) | 749 (33.32) |  | 4479 (27.14) | 801 (37.08) |  |
| **Smoking before pregnancy** |  |  |  |  |  |  |
| No ^c^ | 11621 (70.79) | 1445 (64.28) | <0.001 | 11752 (71.21) | 1314 (60.83) | <0.001 |
| Yes | 4795 (29.21) | 803 (35.72) |  | 4752 (28.79) | 846 (39.17) |  |
| **Smoking during pregnancy** |  |  |  |  |  |  |
| No ^c^ | 11885 (72.40) | 1499 (66.68) | <0.001 | 12025 (72.86) | 1359 (62.92) | <0.001 |
| Yes | 4531 (27.60) | 749 (33.32) |  | 4479 (27.14) | 801 (37.08) |  |
| **Mothers** |  |  |  |  |  |  |
| **Alcohol consumption before pregnancy** |  |  |  |  |  |  |
| No | 15991 (97.41) | 2137 (95.06) | <0.001 | 16089 (97.49) | 2039 (94.40) | <0.001 |
| Yes | 425 (2.59) | 111 (4.94) |  | 415 (2.51) | 121 (5.60) |  |
| **Alcohol consumption during pregnancy** |  |  |  |  |  |  |
| No | 16378 (99.77) | 2233 (99.33) | <0.001 | 16465 (99.76) | 2146 (99.35) | 0.002 |
| Yes | 38 (0.23) | 15 (0.67) |  | 39 (0.24) | 14 (0.65) |  |
| **Smoking before pregnancy** |  |  |  |  |  |  |
| No ^c^ | 16384 (99.81) | 2238 (99.56) | 0.035 | 16473 (99.81) | 2149 (99.49) | 0.006 |
| Yes | 32 (0.19) | 10 (0.44) |  | 31 (0.19) | 11 (0.51) |  |
| **Smoking during pregnancy** |  |  |  |  |  |  |
| No ^c^ | 16410 (99.96) | 2245 (99.87) | 0.147 | 16500 (99.98) | 2155 (99.77) | <0.001 |
| Yes | 6 (0.04) | 3 (0.13) |  | 4 (0.02) | 5 (0.23) |  |
| ^a^ chi-squared test or Fisher's exact test. | | | | | | |
| ^b^ row percentage | | | | | | |
| ^c^ Including non-smokers and former smokers | | | | | | |

| **Supplementary Table S5. Parental alcohol consumption and smoking according to maternal exposure to major life events** | | | | | | |
| --- | --- | --- | --- | --- | --- | --- |
| **Characteristics** | **Maternal exposure to major life events** | | | | | |
|  | **During the year before pregnancy** | | | **During pregnancy** | | |
|  | **Unexposed (N [%])** | **Exposed (N [%])** | ***P* value ^a^** | **Unexposed (N [%])** | **Exposed (N [%])** | ***P* value ^a^** |
| **Total ^b^** | 15653 (83.87) | 3011 (16.13) |  | 16543 (88.64) | 2121 (11.36) |  |
| **Fathers** |  |  |  |  |  |  |
| **Alcohol consumption before pregnancy** |  |  |  |  |  |  |
| No | 13230 (84.52) | 2374 (78.84) | <0.001 | 14001 (84.63) | 1603 (75.58) | <0.001 |
| Yes | 2423 (15.48) | 637 (21.16) |  | 2542 (15.37) | 518 (24.42) |  |
| **Smoking before pregnancy** |  |  |  |  |  |  |
| Never | 10621 (67.85) | 1918 (63.70) | <0.001 | 11290 (68.25) | 1249 (58.89) | <0.001 |
| Former | 420 (2.68) | 107 (3.55) |  | 465 (2.81) | 62 (2.92) |  |
| Current | 4612 (29.46) | 986 (32.75) |  | 4788 (28.94) | 810 (38.19) |  |
| **Smoking during pregnancy** |  |  |  |  |  |  |
| Never | 11020 (70.40) | 2005 (66.59) | <0.001 | 11702 (70.74) | 1323 (62.38) | <0.001 |
| Former | 294 (1.88) | 65 (2.16) |  | 322 (1.95) | 37 (1.74) |  |
| Current | 4339 (27.72) | 941 (31.25) |  | 4519 (27.32) | 761 (35.88) |  |
| **Smoking before pregnancy** |  |  |  |  |  |  |
| No ^c^ | 11041 (70.54) | 2025 (67.25) | <0.001 | 11755 (71.06) | 1311 (61.81) | <0.001 |
| Yes | 4612 (29.46) | 986 (32.75) |  | 4788 (28.94) | 810 (38.19) |  |
| **Smoking during pregnancy** |  |  |  |  |  |  |
| No ^c^ | 11314 (72.28) | 2070 (68.75) | <0.001 | 12024 (72.68) | 1360 (64.12) | <0.001 |
| Yes | 4339 (27.72) | 941 (31.25) |  | 4519 (27.32) | 761 (35.88) |  |
| **Mothers** |  |  |  |  |  |  |
| **Alcohol consumption before pregnancy** |  |  |  |  |  |  |
| No | 15259 (97.48) | 2869 (95.28) | <0.001 | 16140 (97.56) | 1988 (93.73) | <0.001 |
| Yes | 394 (2.52) | 142 (4.72) |  | 403 (2.44) | 133 (6.27) |  |
| **Alcohol consumption during pregnancy** |  |  |  |  |  |  |
| No | 15611 (99.73) | 3000 (99.63) | 0.47 | 16503 (99.76) | 2108 (99.39) | 0.005 |
| Yes | 42 (0.27) | 11 (0.37) |  | 40 (0.24) | 13 (0.61) |  |
| **Smoking before pregnancy** |  |  |  |  |  |  |
| No ^c^ | 15622 (99.80) | 3000 (99.63) | 0.12 | 16516 (99.84) | 2106 (99.29) | <0.001 |
| Yes | 31 (0.20) | 11 (0.37) |  | 27 (0.16) | 15 (0.71) |  |
| **Smoking during pregnancy** |  |  |  |  |  |  |
| No ^c^ | 15646 (99.96) | 3009 (99.93) | 0.97 | 16539 (99.98) | 2116 (99.76) | <0.001 |
| Yes | 7 (0.04) | 2 (0.07) |  | 4 (0.02) | 5 (0.24) |  |
| ^a^ chi-squared test or Fisher's exact test. | | | | | | |
| ^b^ row percentage | | | | | | |
| ^c^ Including non-smokers and former smokers | | | | | | |

| **Supplementary Table S6. Parental alcohol consumption and smoking and autistic behaviors according to paternal exposure to major life events** | | | | | | | | |
| --- | --- | --- | --- | --- | --- | --- | --- | --- |
| **Characteristics** | **Paternal exposure during the three months before the mother's pregnancy** | | | | **Paternal exposure during the mother's pregnancy** | | | |
|  | **Unexposed** | | **Exposed** | | **Unexposed** | | **Exposed** | |
|  | **Total**  **(N [%])** | **Autistic behaviors**  **(N [rate ^a^])** | **Total**  **(N [%])** | **Autistic behaviors**  **(N [rate ^a^])** | **Total**  **(N [%])** | **Autistic behaviors**  **(N [rate ^a^])** | **Total**  **(N [%])** | **Autistic behaviors**  **(N [rate ^a^])** |
| **Fathers** |  |  |  |  |  |  |  |  |
| **Alcohol** **consumption before pregnancy** |  |  |  |  |  |  |  |  |
| No | 13868 (74.30) | 1017 (73.33) | 1736 (9.30) | 161 (92.74) | 13981 (74.91) | 1036 (74.10) | 1623 (8.70) | 142 (87.49) |
| Yes | 2548 (13.65) | 179 (70.25) | 512 (2.74) | 65 (126.95) | 2523 (13.52) | 178 (70.55) | 537 (2.88) | 66 (122.91) |
| **Smoking before pregnancy** |  |  |  |  |  |  |  |  |
| Never | 11173 (59.86) | 821 (73.48) | 1366 (7.32) | 143 (104.69) | 11297 (60.53) | 852 (75.42) | 1242 (6.65) | 112 (90.18) |
| Former | 448 (2.40) | 29 (64.73) | 79 (0.42) | 6 (75.95) | 455 (2.44) | 27 (59.34) | 72 (0.39) | 8 (111.11) |
| Current | 4795 (25.69) | 346 (72.16) | 803 (4.30) | 77 (95.89) | 4752 (25.46) | 335 (70.50) | 846 (4.53) | 88 (104.02) |
| **Smoking during pregnancy** |  |  |  |  |  |  |  |  |
| Never | 11577 (62.03) | 835 (72.13) | 1448 (7.76) | 148 (102.21) | 11714 (62.76) | 865 (73.84) | 1311 (7.02) | 118 (90.01) |
| Former | 308 (1.65) | 26 (84.42) | 51 (0.27) | 7 (137.25) | 311 (1.67) | 26 (83.60) | 48 (0.26) | 7 (145.83) |
| Current | 4531 (24.28) | 335 (73.94) | 749 (4.01) | 71 (94.79) | 4479 (24.00) | 323 (72.11) | 801 (4.29) | 83 (103.62) |
| **Smoking before pregnancy** |  |  |  |  |  |  |  |  |
| No ^b^ | 11621 (62.26) | 850 (73.14) | 1445 (7.74) | 149 (103.11) | 11752 (62.97) | 879 (74.80) | 1314 (7.04) | 120 (91.32) |
| Yes | 4795 (25.69) | 346 (72.16) | 803 (4.30) | 77 (95.89) | 4752 (25.46) | 335 (70.50) | 846 (4.53) | 88 (104.02) |
| **Smoking during pregnancy** |  |  |  |  |  |  |  |  |
| No ^b^ | 11885 (63.68) | 861 (72.44) | 1499 (8.03) | 155 (103.40) | 12025 (64.43) | 891 (74.10) | 1359 (7.28) | 125 (91.98) |
| Yes | 4531 (24.28) | 335 (73.94) | 749 (4.01) | 71 (94.79) | 4479 (24.00) | 323 (72.11) | 801 (4.29) | 83 (103.62) |
| **Mothers** |  |  |  |  |  |  |  |  |
| **Alcohol consumption before pregnancy** |  |  |  |  |  |  |  |  |
| No | 15991 (85.68) | 1161 (72.60) | 2137 (11.45) | 205 (95.93) | 16089 (86.20) | 1178 (73.22) | 2039 (10.92) | 188 (92.20) |
| Yes | 425 (2.28) | 35 (82.35) | 111 (0.59) | 21 (189.19) | 415 (2.22) | 36 (86.75) | 121 (0.65) | 20 (165.29) |
| **Alcohol consumption during pregnancy** |  |  |  |  |  |  |  |  |
| No | 16378 (87.75) | 1192 (72.78) | 2233 (11.96) | 222 (99.42) | 16465 (88.22) | 1208 (73.37) | 2146 (11.50) | 206 (95.99) |
| Yes | 38 (0.20) | 4 (105.26) | 15 (0.08) | 4 (266.67) | 39 (0.21) | 6 (153.85) | 14 (0.08) | 2 (142.86) |
| ^a^ Rate was calculated as the number of preschool children screened positive for autistic behaviors per thousand children in each stratum. | | | | | | | | |
| ^b^ Including non-smokers and former smokers | | | | | | | | |

| **Supplementary Table S7. Parental alcohol consumption and smoking and autistic behaviors according to maternal exposure to major life events** | | | | | | | | |
| --- | --- | --- | --- | --- | --- | --- | --- | --- |
| **Characteristics** | **Maternal exposure during the year before pregnancy** | | | | **Maternal exposure during pregnancy** | | | |
|  | **Unexposed** | | **Exposed** | | **Unexposed** | | **Exposed** | |
|  | **Total**  **(N [%])** | **Autistic behaviors**  **(N [rate ^a^])** | **Total**  **(N [%])** | **Autistic behaviors**  **(N [rate ^a^])** | **Total**  **(N [%])** | **Autistic behaviors**  **(N [rate ^a^])** | **Total**  **(N [%])** | **Autistic behaviors**  **(N [rate ^a^])** |
| **Fathers** |  |  |  |  |  |  |  |  |
| **Alcohol** **consumption before pregnancy** |  |  |  |  |  |  |  |  |
| No | 13230 (70.89) | 980 (74.07) | 2374 (12.72) | 198 (83.40) | 14001 (75.02) | 1041 (74.35) | 1603 (8.59) | 137 (85.46) |
| Yes | 2423 (12.98) | 186 (76.76) | 637 (3.41) | 58 (91.05) | 2542 (13.62) | 198 (77.89) | 518 (2.78) | 46 (88.80) |
| **Smoking before pregnancy** |  |  |  |  |  |  |  |  |
| Never | 10621 (56.91) | 796 (74.95) | 1918 (10.28) | 168 (87.59) | 11290 (60.49) | 854 (75.64) | 1249 (6.69) | 110 (88.07) |
| Former | 420 (2.25) | 27 (64.29) | 107 (0.57) | 8 (74.77) | 465 (2.49) | 31 (66.67) | 62 (0.33) | 4 (64.52) |
| Current | 4612 (24.71) | 343 (74.37) | 986 (5.28) | 80 (81.14) | 4788 (25.65) | 354 (73.93) | 810 (4.34) | 69 (85.19) |
| **Smoking during pregnancy** |  |  |  |  |  |  |  |  |
| Never | 11020 (59.04) | 811 (73.59) | 2005 (10.74) | 172 (85.79) | 11702 (62.70) | 866 (74.00) | 1323 (7.09) | 117 (88.44) |
| Former | 294 (1.58) | 27 (91.84) | 65 (0.35) | 6 (92.31) | 322 (1.73) | 30 (93.17) | 37 (0.20) | 3 (81.08) |
| Current | 4339 (23.25) | 328 (75.59) | 941 (5.04) | 78 (82.89) | 4519 (24.21) | 343 (75.90) | 761 (4.08) | 63 (82.79) |
| **Smoking before pregnancy** |  |  |  |  |  |  |  |  |
| No ^b^ | 11041 (59.16) | 823 (74.54) | 2025 (10.85) | 176 (86.91) | 11755 (62.98) | 885 (75.29) | 1311 (7.02) | 114 (86.96) |
| Yes | 4612 (24.71) | 343 (74.37) | 986 (5.28) | 80 (81.14) | 4788 (25.65) | 354 (73.93) | 810 (4.34) | 69 (85.19) |
| **Smoking during pregnancy** |  |  |  |  |  |  |  |  |
| No ^b^ | 11314 (60.62) | 838 (74.07) | 2070 (11.09) | 178 (85.99) | 12024 (64.42) | 896 (74.52) | 1360 (7.29) | 120 (88.24) |
| Yes | 4339 (23.25) | 328 (75.59) | 941 (5.04) | 78 (82.89) | 4519 (24.21) | 343 (75.90) | 761 (4.08) | 63 (82.79) |
| **Mothers** |  |  |  |  |  |  |  |  |
| **Alcohol consumption before pregnancy** |  |  |  |  |  |  |  |  |
| No | 15259 (81.76) | 1129 (73.99) | 2869 (15.37) | 237 (82.61) | 16140 (86.48) | 1197 (74.16) | 1988 (10.65) | 169 (85.01) |
| Yes | 394 (2.11) | 37 (93.91) | 142 (0.76) | 19 (133.80) | 403 (2.16) | 42 (104.22) | 133 (0.71) | 14 (105.26) |
| **Alcohol consumption during pregnancy** |  |  |  |  |  |  |  |  |
| No | 15611 (83.64) | 1160 (74.31) | 3000 (16.07) | 254 (84.67) | 16503 (88.42) | 1232 (74.65) | 2108 (11.29) | 182 (86.34) |
| Yes | 42 (0.23) | 6 (142.86) | 11 (0.06) | 2 (181.82) | 40 (0.21) | 7 (175.00) | 13 (0.07) | 1 (76.92) |
| ^a^ Rate was calculated as the number of preschool children screened positive for autistic behaviors per thousand children in each stratum. | | | | | | | | |
| ^b^ Including non-smokers and former smokers | | | | | | | | |

| **Supplementary Table S8. Association between parental major life events and autistic behaviors in preschool children stratified by the sex of the child** | | | | | | | |
| --- | --- | --- | --- | --- | --- | --- | --- |
| **Parental exposure to major life events** | **Boys** | | | **Girls** | | | ***P* for interaction** |
|  | **Total (N [%])** | **Autistic behaviors (N [rate ^a^])** | **OR (95%CI) ^b^** | **Total (N [%])** | **Autistic behaviors (N [rate ^a^])** | **OR (95%CI) ^b^** |  |
| **Fathers** |  |  |  |  |  |  |  |
| **During the three months before the mother's pregnancy** |  |  |  |  |  |  |  |
| Unexposed | 8835 (47.34) | 720 (81.49) | ref. | 7581 (40.62) | 476 (62.79) | ref. |  |
| Exposed | 1164 (6.24) | 134 (115.12) | 1.36 (1.12, 1.66) | 1084 (5.81) | 92 (84.87) | 1.29 (1.02, 1.63) | 0.73 |
| **During the mother's pregnancy** |  |  |  |  |  |  |  |
| Unexposed | 8854 (47.44) | 728 (82.22) | ref. | 7650 (40.99) | 486 (63.53) | ref. |  |
| Exposed | 1145 (6.13) | 126 (110.04) | 1.26 (1.03, 1.54) | 1015 (5.44) | 82 (80.79) | 1.21 (0.95, 1.55) | 0.82 |
| **Mothers** |  |  |  |  |  |  |  |
| **During the year before pregnancy** |  |  |  |  |  |  |  |
| Unexposed | 8453 (45.29) | 704 (83.28) | ref. | 7200 (38.58) | 462 (64.17) | ref. |  |
| Exposed | 1546 (8.28) | 150 (97.02) | 1.11 (0.92, 1.34) | 1465 (7.85) | 106 (72.35) | 1.07 (0.86, 1.34) | 0.84 |
| **During pregnancy** |  |  |  |  |  |  |  |
| Unexposed | 8885 (47.61) | 753 (84.75) | ref. | 7658 (41.03) | 486 (63.46) | ref. |  |
| Exposed | 1114 (5.97) | 101 (90.66) | 0.98 (0.79, 1.23) | 1007 (5.40) | 82 (81.43) | 1.21 (0.95, 1.56) | 0.21 |
| OR = Odds Ratio, CI = Confidence Interval | | | | | | | |
| ^a^ Rate was calculated as the number of preschool children screened positive for autistic behaviors per thousand children in each stratum. | | | | | | | |
| ^b^ For parental major life events before the mother's pregnancy, models were adjusted for maternal and paternal age at the child's birth, educational level, occupations, maternal mode of conception, parity, and the child's sex. For parental major life events during the mother's pregnancy, models were further adjusted for parental diseases and medication use before the mother's pregnancy. Both models were conducted after multiple imputations. | | | | | | | |
